# Targeting 17β-estradiol biosynthesis in neural stem cells improves stroke outcome

**DOI:** 10.3389/fncel.2022.917181

**Published:** 2022-07-22

**Authors:** Shalmali Patkar, Dafe Uwanogho, Michel Modo, Rothwelle J. Tate, Robin Plevin, Hilary V. O. Carswell

**Affiliations:** ^1^Strathclyde Institute of Pharmacy and Biological Sciences, University of Strathclyde, Glasgow, United Kingdom; ^2^Department of Neuroscience, James Black Centre, King’s College London, London, United Kingdom; ^3^Department of Radiology, University of Pittsburgh, Pittsburgh, PA, United States

**Keywords:** cerebral ischemia, estrogen, steroidogenesis, cell therapy, transplantation, neuroprotection, brain repair

## Abstract

Dax-1 (dosage-sensitive sex reversal adrenal hypoplasia congenital region on X-chromosome gene 1) blocks 17β-estradiol biosynthesis and its knockdown would be expected to increase 17β-estradiol production. We hypothesized that knockdown of Dax-1 in a conditionally immortalized neural stem cell (NSC) line, MHP36, is a useful approach to increase 17β-estradiol production. Short hairpin (sh) RNA targeted to *Dax-1* in NSCs, namely MHP36-Dax1KD cells, resulted in the degradation of *Dax-1* RNA and attenuation of Dax-1 protein expression. *In vitro*, MHP36-Dax1KD cells exhibited overexpression of aromatase and increased 17β-estradiol secretion compared to MHP36 cells. As 17β-estradiol has been shown to promote the efficacy of cell therapy, we interrogated the application of 17β-estradiol-enriched NSCs in a relevant *in vivo* disease model. We hypothesized that MHP36-Dax1KD cells will enhance functional recovery after transplantation in a stroke model. C57BL/6 male adult mice underwent ischemia/reperfusion by left middle cerebral artery occlusion for 45 min using an intraluminal thread. Two days later male mice randomly received vehicle, MHP36 cells, MHP36-Dax1KD cells, and MHP36 cells suspended in 17β-estradiol (100 nm) or 17β-estradiol alone (100 nm) with serial behavioral testing over 28 days followed by post-mortem histology and blinded analysis. Recovery of sensorimotor function was accelerated and enhanced, and lesion volume was reduced by MHP36-Dax1KD transplants. Regarding mechanisms, immunofluorescence indicated increased synaptic plasticity and neuronal differentiation after MHP36-Dax1KD transplants. In conclusion, knockdown of Dax-1 is a useful target to increase 17β-estradiol biosynthesis in NSCs and improves functional recovery after stroke *in vivo*, possibly mediated through neuroprotection and improved synaptic plasticity. Therefore, targeting 17β-estradiol biosynthesis in stem cells may be a promising therapeutic strategy for enhancing the efficacy of stem cell-based therapies for stroke.

## Introduction

Ischemic stroke is a major cardiovascular disease that causes 5 million deaths with a further 5 million people being permanently disabled worldwide every year. Stem cell technology is aimed at accelerating and augmenting functional recovery after stroke (Hermann et al., 2014). In animal models of ischemic stroke mesenchymal stem cells, neural stem cells (NSCs) and induced pluripotent stem cells have been shown to improve recovery and infarct size (for systematic review see Zhang et al., 2021). A “first in man study”, the Pilot Investigation of Stem Cells in Stroke (PISCES) clinical trials show the feasibility of intracerebral implantation of human neural stem cells (NSCs) after stroke with improvements shown in residual but not absent upper limb function at baseline in their phase two trials (Muir et al., 2020). However, increasing evidence suggests we need to increase the therapeutic efficacy of the transplanted cells to enhance stroke recovery (Hermann et al., 2014; Damian et al., 2021). These improvements may be achieved by manipulating17β-estradiol (Liang et al., 2016; Li et al., 2020; Mihai et al., 2021). 17β-estradiol has beneficial effects on various stem cells and progenitor cells in improving recovery after myocardial ischemia (Yuan et al., 2018) and cerebral ischemia (Liang et al., 2016). In addition, 17β-estradiol has neuroprotective and neuroregenerative effects in experimental cerebral ischemia (Hurn and Macrae, 2000; Horsburgh et al., 2002; Liu et al., 2007; Etgen et al., 2011; Lu et al., 2020). Some of these effects may be mediated by 17β-estradiol-induced expression of growth factors and their receptors (e.g., insulin-like growth factor-1, Sohrabji, 2015). However beneficial effects of 17β-estradiol are not translated clinically and may even worsen stroke (Wassertheil-Smoller et al., 2003). Detrimental effects are proven in preclinical stroke models (Carswell et al., 2004; Bingham et al., 2005; Strom et al., 2011), potentially due to systemic 17β-estradiol treatment that is too late after menopause (Hodis et al., 2016; Guo et al., 2020) or that is supraphysiological (Carswell et al., 2009; Strom et al., 2009). Therefore, to harness beneficial 17β-estradiol effects, while circumventing potential detrimental systemic effects, we genetically modified NSCs to overexpress 17β-estradiol.

Maudsley hippocampal murine neural progenitor line clone 36 (MHP36) cells (Sinden et al., 1997) migrate to injury sites (Ransohoff, 2007), functionally integrate into the damaged brain (Gray et al., 2000), promote brain repair (Lee et al., 2010), and restore cognitive and functional deficits (Gray et al., 2000; Modo et al., 2002; Patkar et al., 2012) after various types of neurological damage including stroke. Importantly, MHP36 cells exhibit temperature-sensitive control over cell division at body temperature and have not given rise to tumors months after *in vivo* engraftment in any of the studies (e.g., Gray et al., 2000). In terms of therapeutic value, human equivalents to MHP36 cells, conditionally immortalized using mycER, are currently in phase two clinical trials for stroke (Muir et al., 2020). Therefore, MHP36 cells would not be considered equivalent to an *in vivo* population of NSCs but have potential as therapeutic vehicles to mediate the repair of neurological damage. It is precisely these above-mentioned properties that make MHP36 cells, a *bona fide* murine NSC line, amiable to genetic manipulation to enhance their therapeutic efficacy.

Dax-1 [dosage-sensitive sex reversal, adrenal hypoplasia congenita (AHC) critical region on the X chromosome, gene 1] is a negative regulator of steroidogenesis. Dax-1 represses the trans-activation of the *aromatase* gene (Wang et al., 2001) which encodes the enzyme that converts testosterone to 17β-estradiol. Therefore, the knockdown of Dax-1 in MHP36 cells (MHP36-Dax1KD) is expected to raise aromatase expression and increase 17β-estradiol production (Patkar et al., 2009; Patkar, 2010). We hypothesize that Dax-1 knockdown by short hairpin RNA targeted to *Dax-1* is a potential target for increasing 17β-estradiol secretion in stem cells *in vitro*. Further, taking advantage of stem cells’ innate capacity to migrate to sites of injury to deliver sustained 17β-estradiol levels locally to the brain lesion, we hypothesize that 17β-estradiol overexpressing NSCs will improve functional recovery after stroke compared to non-modified NSCs and/or 17β-estradiol *in vivo*. We report *in vitro* on the characterization of MHP36-Dax1KD cells for 17β-estradiol and *in vivo* on sensorimotor functional recovery and mechanistic insight after MHP36-Dax1KD transplants.

## Materials and Methods

### Derivation and culturing of NSCs line

MHP36 cells were derived from the H-2Kb-tsA58 transgenic embryonic mouse hippocampal neuroepithelium (Sinden et al., 1997). Therefore, *in vitro* at the permissive temperature of 33°C, MHP36 cells may be maintained and expanded in an undifferentiated state. However, *in vivo* (after transplantation) at temperatures 37–39°C, MHP36 cells cease to divide, hence reducing the risk of tumor formation *in vivo*, and entering the pathway to differentiation (Gray et al., 2000). MHP36 and MHP36-Dax1KD were cultured from frozen stocks and maintained at 33°C in DMEM/F12 (Gibco, UK) with the various additives as previously described (Patkar et al., 2012).

### Genetic modification of NSCs to overexpress 17β-estradiol and their characterization

A lentiviral vector system was used for silencing *Dax-1*. The insert was a short hairpin RNA (shRNA) targeted to *Dax-1* (Mission RNAs from Sigma) which resulted in the degradation of *Dax-1* RNA and, in turn, the attenuation of Dax-1 protein expression. Lentiviral particles, made using standard protocols, were used to transduce NSCs at various multiplicity of infection. Stably expressing MHP36 cells were then selected using 10 μg/ml puromycin (Sigma-Aldrich, UK). The level of expression for Dax-1 and aromatase were determined using immunofluorescence and Western blotting and the production of 17β-estradiol was determined using commercially available estradiol ELISA kit *in vitro* (DRG International Inc., USA). Immunofluorescence, Western blotting, and RT-PCR were used to show the presence of estrogen receptors (*n* = 3 independent experiments per group).

### Immunofluorescent staining of MHP36 and MHP36-Dax1KD cells

MHP36 and MHP36-Dax1KD cells were grown on fibronectin-coated cover slips and fixed in 4% paraformaldehyde (PFA) in phosphate-buffered saline (PBS) for 10 min then in cold methanol for 10 min. Non-specific binding was blocked (1% (w/v) bovine serum albumin (BSA) in PBS for 1 h at room temperature). The primary antibodies used were: rabbit anti-aromatase (1:100, gift from J. Hutchison and L. Garcia-Segura, Spain), mouse anti-ERα (1:100, Novachem, UK), mouse anti-ERβ (1:50, CO1531, gift from G. Greene), rabbit anti-GPR30 (1:200, Abcam, UK), and rabbit anti-Dax-1 (1:200, Abcam, UK). After overnight incubation at 4°C, secondary antibodies raised against rabbit IgG bound to Texas red (Vector labs), mouse IgG bound to Alexafluor 555 (Chemicon, UK), and mouse IgG bound to fluorescein isothiocyanate (Sigma-Aldrich, UK) at a dilution of 1:100 in PBS for 1 h were used before mounting with Vectashield with DAPI (Vector Labs, UK) then viewed and photographed using a Nikon Eclipse E600 Oil Immersion microscope connected to a photometrics (CoolSnapFx) digital camera managed by MetaMorph software (Molecular Devices, UK). A negative control was included in every run which included no primary antibody incubation. The acquired images were analyzed using standard procedures, including spatial calibration, image acquisition, and thresholding. MCF7 cells were used as positive controls for ERα and GPR30.

### Western blotting of MHP36 and MHP36-Dax1KD cells

Whole cell extracts were prepared by harvesting cells in 1× loading buffer (2% w/v SDS, 50 mm dithiothreitol, 10% (v/v) glycerol, 63 mm Tris-HCl (pH 6.8), 5 mm EDTA, and 2 mm NaP_2_O_7_, 0.007% w/v bromophenol blue). Proteins were separated on a 10% Medium polyacrylamide denaturing gel using a running buffer for 90 min at a constant voltage (120 V), electrophoretically transferred using transfer buffer to a nitrocellulose membrane (Amersham Pharmacia Biotech, UK) for 90 min at a constant current (300 mA). The membrane was blocked for 90 min at room temperature with 2% (w/v) BSA made in NATT buffer and then probed with the primary antibody in 0.2% (w/v) BSA in NATT, overnight. After being washed in NATT for 90 min, the membrane was incubated for 90 min with a specific secondary antibody (Peroxidase-AffiniPure Goat Anti-Rabbit IgG, Donkey Anti-Mouse IgG, Stratech Scientific Ltd, UK) at 1:7,500 dilution in 0.2% BSA in NATT. The signal was developed by enhanced chemiluminescence (Amersham Pharmacia Biotech, UK) and visualized on a Kodak BioMax film. Blots were stripped and re-probed with anti-GAPDH (AbCam, UK) or total anti-p38 (SantaCruz Biotechnology, UK) antibody at 1:15,000/1:10,000 dilutions as an internal control for loading, respectively. Primary antibodies used: Rabbit against- aromatase, Dax-1, GPR30; Mouse against- ERα (sources detailed above). MCF7 cells were used as validation controls, run across multiple blots.

### Reverse Transcriptase (RT)-PCR of MHP36 and MHP36-Dax1KD cells

RNA was extracted from MHP36 and MHP36-Dax1KD cells using the GenElute^TM^ Mammalian Total RNA Miniprep Kit (Sigma-Aldrich, UK) according to the manufacturer’s instructions. First-strand synthesis was carried out using Superscript III (Invitrogen) with a NV-Clamped Oligo d(T)18 primer. The PCR primers were designed in-house and were as follows: *ERα* (sense—5’-AAT TCT GAC AAT CGA CGC CAG-3’; antisense—5’-GTG CTT CAA CAT TCT CCC TCC TC-3’); *ERβ* (sense—5’-CTT GGT CAC GTA CCC CTT AC-3’; antisense—5’-GTA TCG CGT CAC TTT CCT TT-3’); *GPR30* (sense—5’-CCT TAA GCT GCT GGA ATT GTG G-3’; antisense—5’-GCC GCC AGG TTG ATG AAG TAC-3’). Endpoint RT-PCR was performed by using HotStarTaq Plus Master Mix Kit (Qiagen). The RT-PCR protocol had an initial activation step of 5 min at 95°C followed by 30 cycles at 94°C for 1 min, 60°C for 30 s, and 72°C for 1 min and was performed on a Primus-96 thermal cycler (MWG-Biotech, UK). Amplicons underwent 2% (w/v) tris-boric acid-EDTA agarose gel electrophoresis with ethidium bromide staining and visualized under UV. The PCR products were gel isolated using the QIAquick Gel Extraction kit (Qiagen), and sequenced in both directions using BigDye Terminator v3.1 Chemistry (Applied Biosystems, UK) and a 3100-Avant Genetic Analyzer (Applied Biosystems, UK). Querying the GenBank database with the obtained sequences provided confirmation of the identity of the amplicons. Ovary and testis are used as positive controls for estrogen receptors.

### ELISA of MHP36 and MHP36-Dax1KD cells and supernatants

The assay used to measure 17β-estradiol levels was designed to analyze human plasma. The steroid releasing agent was not designed to remove rodent binding proteins. Therefore, the 17β-estradiol was first extracted from the NSCs or supernatant (media) after 48 h of plating and reconstituted in steroid-free human serum (DRG International Inc., USA) before using the 17β-estradiol immunoassay (ELISA, DRG International Inc., USA). Briefly, to 200 μl of the sample, 800 μl of methanol was added to an Eppendorf and subsequently vortexed for 1 min. The samples were then centrifuged at 10,000 rpm for 5 min. The supernatant was vacuum-dried (Savant Instruments Inc., USA) until only a white residue (the 17β-estradiol) remained (5 h). This was reconstituted with 100 μl of steroid-free human serum and vortexed thoroughly. The sample was now 2.0 times more concentrated and was ready to be measured using the ELISA kit. Mouse plasma (female 129svJ mouse) was used as a positive control.

### Surgery for MCAO, laser doppler flowmetry, grafting, and outcome measures

Twenty-four male C57BL/6 mice (Charles River, UK) (12–14 weeks old, 25–30 g) were used, housed in a controlled environment with a 12:12 h light cycle beginning at 06:00 and temperature maintained at 22°C and allowed *ad libitum* access to food and water. The experiment complied with the UK Animals (Scientific) Procedures Act (1986) with approval by the Home Office of the United Kingdom (Project License number 60/4469) and Ethical Review of the University of Strathclyde and in adherence with ARRIVE guidelines (Percie du Sert et al., 2020).

Following completion of baseline testing, focal ischemia was induced within the left hemisphere by transient (45 min) middle cerebral artery occlusion (MCAO) using the intraluminal thread model, an occlusion time we have previously found to exhibit no mortalities (Patkar et al., 2012). Briefly, mice were anesthetized with 3% isoflurane (Bimeda-MTC Animal Health Incorporated, UK) mixed with 1% oxygen and maintained with 1.5 ± 0.25% isoflurane. The core body temperature was regulated at 37 ± 0.5°C and a 7–0 silicone monofilament (Doccol Ltd., UK) was introduced into the external carotid artery and advanced along the internal carotid artery until occluding the origin of the MCA. Animals were maintained on 1.25% isoflurane during the occlusion time. After 45 min, the filament was withdrawn to establish reperfusion, an occlusion duration shown in a pilot study to induce cortical as well as striatal damage. Sham-operated mice underwent the same procedure except the filament was not advanced along the internal carotid artery.

In each animal, laser Doppler flowmetry (Moor Instruments, UK) was used to monitor cerebral blood flow (CBF) continuously, before and during MCAO as well as during reperfusion. Briefly, a small incision of the skin overlying the temporalis muscle was made and a 0.7 mm, flexible, laser Doppler probe (model P10; Moor Instruments, UK) was positioned on the superior portion of the temporal bone and secured with glue. This position corresponded to the MCA territory. Animals were included only when CBF was reduced by ≥85% during ischemia, and successful reperfusion was subsequently achieved. No animals were excluded.

Two days post-MCAO mice were randomly allocated to receive ipsilateral cortical and striatal grafts of vehicle (1 mm N-acetyl-l-cysteine plus 0.0001% v/v ethanol; *n* = 4), MHP36 (*n* = 4), MHP36-Dax1KD (*n* = 6), MHP36 cells suspended in 17β-estradiol [MHP36+E2 (100 nm; *n* = 6) or 17β-estradiol alone (100 nm; *n* = 4)], based on previous pilot experiments. The vehicle was used to suspend cells and for the final dilution of 17β-estradiol to 100 nm, a concentration previously shown to improve stem cell efficacy (Yuan et al., 2018). Before grafting, cells were labeled with the membrane-bound fluorescent marker PKH26 (Sigma, UK), as previously described (Modo et al., 2002). PKH26 has been found to be a reliable marker of transplanted cells (Nicholls et al., 2017). Labeled cells were suspended in 1 mm N-acetyl-l-cysteine (Sigma-Aldrich, UK) plus 0.0001% v/v ethanol in HBSS without Ca^2+^ or Mg^2+^ at a concentration of 25,000 cells/μl. Viability was assessed using trypan blue exclusion in a hemocytometer. Pre-graft viability averaged 90% and post-graft viability averaged 78%. Mice were anesthetized with isoflurane (3% induction, 1.5% maintenance) and mounted on a stereotaxic frame. A 2-μl Hamilton syringe with a 32-gauge beveled needle was descended (Medial/Lateral + 2 mm, Anterior/Posterior -0.26 mm relative to Bregma) through a burr hole to a depth of -1.5 mm (cortical graft) from the surface of the brain. Cell suspension or vehicle (0.5 μl) was injected over 2 min and the syringe was left in place for another 2 min then a second injection was made at depth of -3 mm (striatal graft). A total of 25,000 cells were implanted into each animal. The 48-h time is when the lesion has, at least almost, fully evolved (Rewell et al., 2017). From this time point, alterations in plasticity can be promoted by stem cells resulting in the recovery of function in previous studies (Patkar et al., 2012). Therefore, this time point was chosen as it is deemed to be late enough to limit stem cell effects on evolving lesion and to allow recovery time between surgeries, and early enough to promote brain plasticity to allow us to observe effects on recovery of function.

### Behavioral tests

The mice were handled extensively and habituated to the testing location and apparatus prior to training and testing. Baseline testing was completed 2 days prior to MCAO. Video recordings of the foot fault (ladder rung) test and spontaneous forelimb (cylinder) test were performed by a blinded investigator pre-MCAO and at 2, 7, 14, and 28 days post-MCAO. Foot fault tests have commonly been used as an efficient and sensitive test strategy for chronic assessment of skilled fore- and hind-limb stepping in mice. Mice were tested on the ladder as previously described (Patkar et al., 2012) and each step was scored according to the quality of limb placement based on the scale adapted from Metz and Whishaw (2002). The cylinder test detects even mild asymmetries while factoring out confounding variables such as an overall decrease in activity as additional trials can be performed. Mice were tested in the cylinder as previously described (Schallert et al., 2000) and the laterality score was computed as follows: (# of right only − # of left only)/(# of right only + # of left only + # of both). Normal uninjured performance, for an animal with no preference for right or left forelimb, is at or near zero. Additional two-min trials were performed until at least 10 rearing observations were made. The mice were filmed with a high-speed Panasonic digital camcorder (30 frames/s; shutter speed of 1/1,000). The digital videotapes were analyzed using an HP Pavilion DV2000 laptop. Single frames were imported from the digital video records using a Windows media player on a Windows operating system. For performance of behavior and in all analysis the experimenter was blinded to which treatment mice received.

### Histology and immunofluorescence

After 28 days, mice were perfused through the heart with 0.9% w/v physiological saline followed by 4% PFA. The brains were removed and cryo-protected in a 30% w/v sucrose solution for 48 h and sectioned into 20 μm thick sections.

For lesion volume measurement, eight coronal levels were selected from the mouse atlas (-3.78, -2.75, -1.75, -1.05, -0.38, +0.145, +0.945 and +2.2 mm with respect to Bregma) and stained with hematoxylin and eosin. Areas of ischemic damage were delineated on to scale diagrams representing the eight coronal levels and then measured by means of an MCID image-analysis system. Total volume (mm^3^) of ischemic damage was calculated by the integration of areas (mm^2^) with the distance between each coronal level (mm) with 2.9 mm (rostral limit) and -4.9 mm (caudal limit) with respect to Bregma as the end points.

For immunostaining, three animal brains were randomly selected where possible to avoid bias selection and serial coronal sections stained for the different immunostaining markers. Region of interest (ROI) consisted of the ipsilateral striatum and somatosensory cortex within each brain and for synaptophysin also consisted of the corresponding contralateral ROIs (Bregma 0.3 ± 0.1 mm). Two images were taken in each ROI for quantification. No comparisons were made between the cortex and striatum. The images were acquired using a Leica Epi-fluorescence microscope (Leica Microsystems, Germany) with 200×, 400×, and 100× oil-immersion objectives connected to Metamorph-Pro software. During image acquisition, the threshold and gain on the confocal laser microscope were set using the control for each run of staining. This helped to subtract the background fluorescence. The average intensity in the ROI per area of field of view for mouse anti-glial fibrillary acidic protein (GFAP, Sigma, UK), mouse anti-2’,3’-Cyclic Nucleotide 3’-Phosphodiesterase (CNPase, Chemicon, UK), goat anti-IBA-1 (Abcam, UK) and mouse anti-synaptophysin (Syn, Abcam, UK) was measured using Image J software (Schneider et al., 2012). The obtained values used for graphical representation are a ratio of intensity and area. Percentages of neuronal differentiation (chicken anti-microtubule-associated protein (MAP-2, Chemicon, UK)) and synaptic plasticity (rabbit anti-GAP-43 (growth-associated protein-43), Abcam, UK) were determined by counting the number of cells co-localizing with PKH26 to the total number of PKH26-positive cells within the same field (Nicholls et al., 2017). In all analysis, the experimenter was blinded to which treatment mice received.

#### Statistical analysis

Statistical comparisons in cylinder and ladder rung tests were made using repeated measures ANOVA. Statistical comparisons for all other analyses were done by two-way ANOVA with a *post-hoc* Bonferroni’s test to correct for multiple comparisons or an unpaired *t*-test where two groups were compared. A *p*-value of less than 0.05 was chosen as the significance level for all statistical analyses. All data are presented as mean ± standard error of the mean.

## Results

### MHP36-Dax1KD cells exhibited increased 17β-estradiol synthesis *in vitro*

**Figure 1:**
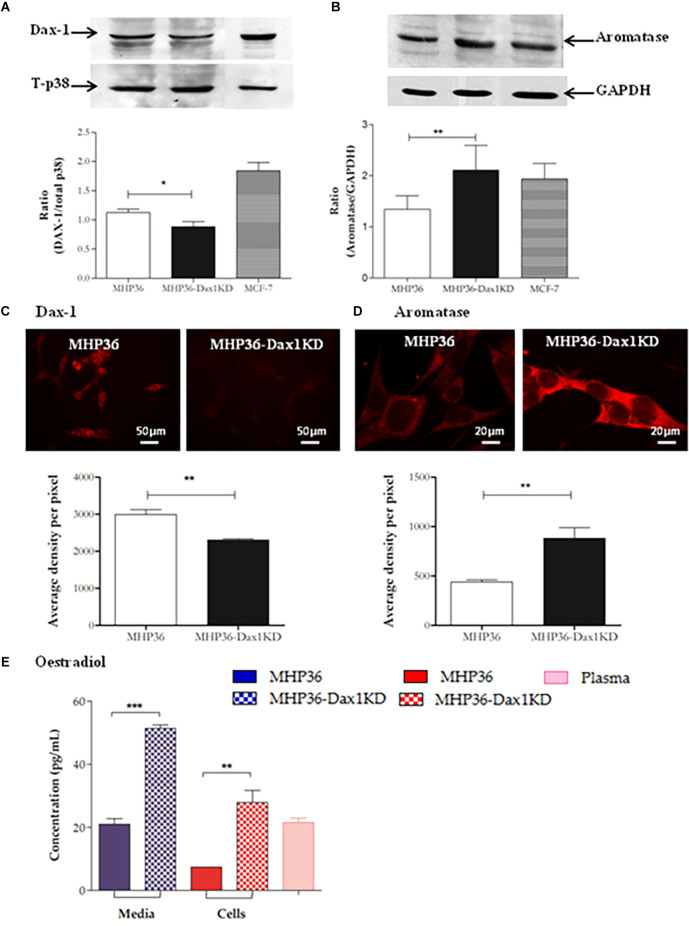
Characterization of MHP36-Dax1KD for 17β-estradiol. Knockdown of expression of Dax-1 and overexpression of aromatase in MHP36-Dax1KD when compared to MHP36 cells using **(A,B)** Western blotting (graphical representation expressed as a ratio over total p38 or GAPDH) and **(C,D)** immunofluorescence (graphical representation expressed as mean intensity per pixel of protein of interest). **(E)** Levels of 17β-estradiol released into the media and produced by MHP36 and MHP36-Dax1KD cells using ELISA with plasma (female 129svJ mouse) as a positive control to show alignment with physiological levels (*p < 0.05, **p < 0.01, ***p < 0.001, unpaired *t*-test, *n* = 3).

**Figure 2:**
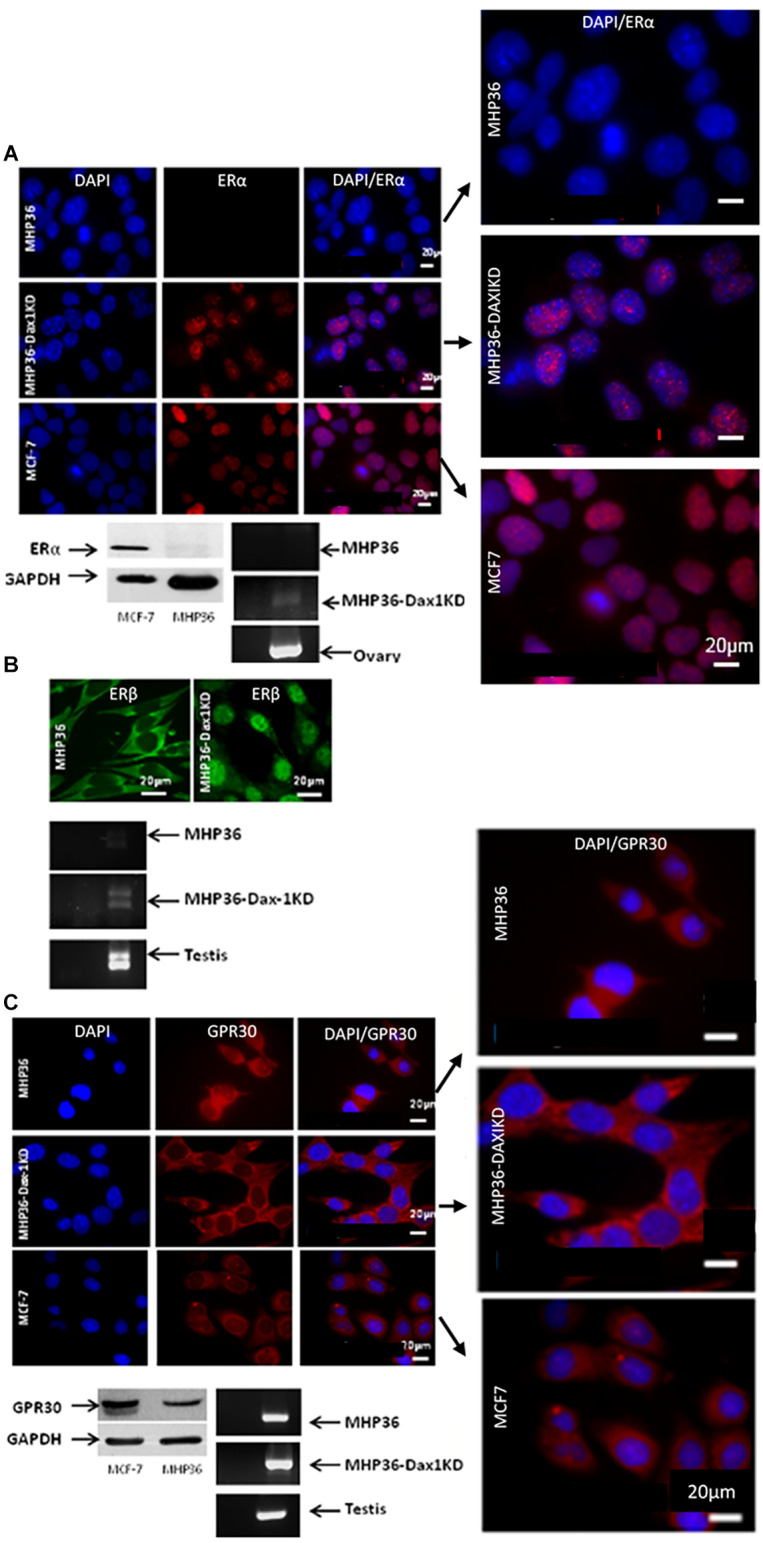
Characterizing the MHP36 and MHP36-Dax1KD for estrogen receptors (ER). **(A)** Immunofluorescence staining (top panel) for ERα in MHP36, MHP36-Dax1KD, and MCF-7 cells (positive for ERα; Bars: 20 μm), with co-localization shown in enlarged images. Western blotting (bottom left panel) and RT-PCR (bottom right panel) confirming immunostaining results, ovary used as positive for ERα. **(B)** Immunofluorescence staining (top panel) for ERβ in MHP36 (cytoplasmic staining) and MHP36-Dax1KD (nuclear staining) cells (Bars: 20 μm). RT-PCR (bottom panel) confirming immunostaining results, testis used as positive for ER*β.* **(C)** Immunofluorescence staining (co-localization shown in enlarged images) and Western blotting (top and bottom left panel) for GPR30 in MHP36, MHP36-Dax1KD, and MCF-7 cells (positive for GPR30; Bars: 20 μm). RT-PCR (bottom right panel) confirming immunostaining results, testis used as positive for GPR30 (representative images of *n* = 3).

A significant decrease in Dax-1 expression and a concomitant significant increase in aromatase expression were achieved in MHP36-Dax1KD when compared to MHP36 confirming successful Dax-1 knockdown (Figure 1). Levels of 17β-estradiol were significantly increased in the media and cells in MHP36-Dax1KD when compared to MHP36 bringing levels from low to high physiological (Figure 1). Immunostaining indicated that MHP36 cells expressed GPR30 and ERβ, but negligible ERα which was supported by Western blots. MHP36-Dax1KD displayed an increase in ERα and ERβ but not GPR30 compared to MHP36, indicated by immunostaining and RT-PCR bands (Figure 2).

### MHP36-Dax1KD grafts completely restored spontaneous forelimb use symmetry

**Figure 3:**
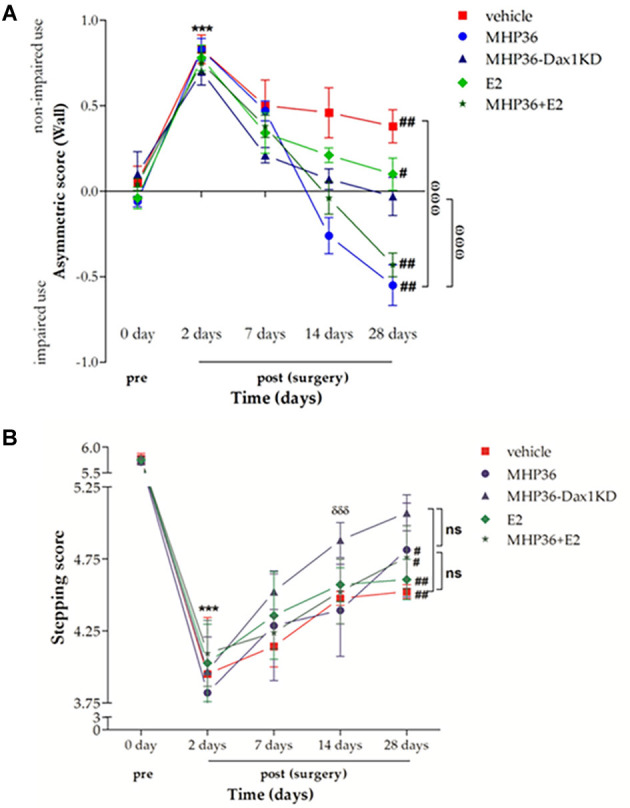
MHP36-Dax1KD grafts restored functional recovery using spontaneous forelimb use asymmetry test and foot fault test. **(A)** A significant asymmetry on cylinder testing at 2 days post-MCAO was observed in all groups. MHP36-Dax1KD improved the functional recovery to pre-MCAO score (***p < 0.001 pre-MCAO vs. 2 days post-MCAO in all groups; ^#^*p* < 0.05, ^##^*p* < 0.01 pre-MCAO vs. 28 days,^ωωω^*p* < 0.001 MHP36 and MHP36+E2 vs. vehicle and MHP36-Dax1KD at 28 days post-MCAO, repeated measures ANOVA; *n* ≥ 4). **(B)** A significant stepping error, on the ladder rung test, at 2 days post-MCAO was observed in all groups. MHP36-Dax1KD grafts not only improved recovery at 28 days (n.s. vs. pre-MCAO) but also accelerated recovery of limb function at 14 days (*** *p* < 0.001 pre-MCAO vs. 2 days post-MCAO in all groups; ^#^*p* < 0.01, ^##^*p* < 0.001 pre-MCAO vs. 28 days post-MCAO; δδδ *p* < 0.001 2 days post-MCAO vs. 14 days post-MCAO; repeated measures ANOVA; vehicle *n* = 4, MHP36 *n* = 4, MHP36-Dax1KD *n* = 6, MHP36+E2 *n* = 6, E2 *n* = 4).

In the cylinder test, there was a significant preference for ipsilateral (non-impaired) forelimb usage in MCAO animals at 2 days post-MCAO when compared to pre-MCAO scores in all injection groups (Figure 3A). Significant preferences for non-impaired forelimb were still evident at 28 days post-MCAO after vehicle or 17β-estradiol (E2). A highly significant preference for the contralateral (“impaired”) forelimb was observed at 28 days post-MCAO after MHP36 **(laterality score = -0.55 ± 0.12)** or MHP36 plus E_2_ **(-0.43 ± 0.035)**, whereas complete restoration of spontaneous forelimb use symmetry to pre-MCAO baseline scores was achieved by 28 days post-MCAO after MHP36-Dax1KD grafts **(-0.03 ± 0.12, n.s.)**.

### MHP36-Dax1KD grafts accelerated and restored limb function

In the ladder test, there was a significant increase in placement errors of the contralateral (“impaired”) forelimb in all groups (Figure 3B) at 2 days post-MCAO when compared to pre-MCAO scores. A significant deficit was still evident at 28 days post-MCAO in all groups except after MHP36-Dax1KD transplantation. Restoration of function was observed by 28 days post-MCAO after MHP36-Dax1KD treatment **(5.07 ± 0.125)** when compared to pre-MCAO scores **(5.73 ± 0.081, n.s.)**. In addition, recovery of limb function was accelerated (observed at 14 days post-MCAO) after MHP36-Dax1KD treatment (*p* < 0.001).

### MHP36-Dax1KD grafts reduced lesion size, promoted plasticity and integration in the host brain

**Figure 4:**
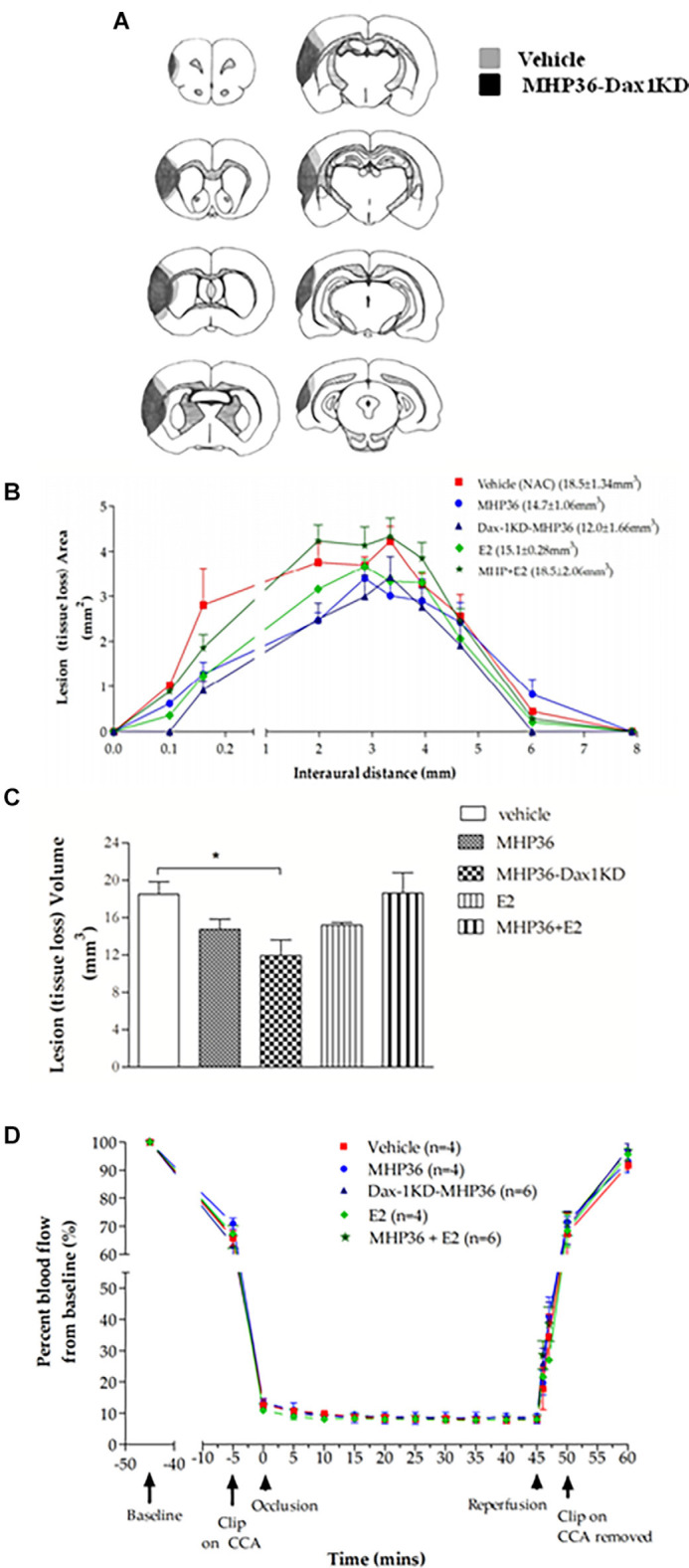
MHP36-Dax1KD grafts reduced lesion size at 28 days post-MCAO with no differences in cerebral perfusion at the time of MCAO. **(A)** Median representatives of the topography of lesion in animals receiving either vehicle or MHP36-Dax1KD represented as shaded area on line diagrams of eight coronal levels. **(B)** Graphical representation of the topography of lesion over the eight coronal levels. **(C)** Bar graph representing lesion volumes measured in all experimental groups. A significant decrease in lesion volume was observed between MHP36-Dax1KD and vehicle treated (*p < 0.01, one-way ANOVA, *post-hoc* Bonferroni’s test to correct for multiple comparisons). **(D)** Cortical blood flow before, during and after MCAO, using laser Doppler flowmetry, presented as a percentage change from baseline blood flow (100%) (CCA = common carotid artery; vehicle *n* = 4, MHP36 *n* = 4, MHP36-Dax1KD *n* = 6, MHP36+E2 *n* = 6, E2 *n* = 4).

**Figure 5:**
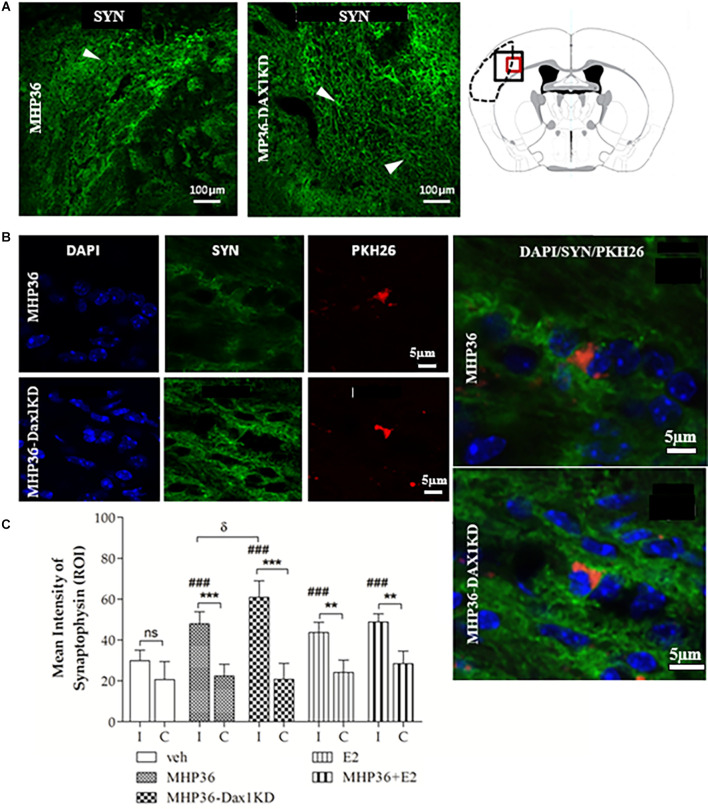
MHP36-Dax1KD grafts increased expression of synaptophysin in the host brain. **(A)** Peri-infarct region of interest in the MHP36 and MHP36-Dax1KD injected animals stained with synaptophysin (scale bar = 100 μm) and schema of the coronal level at Bregma: -0.3 ± 0.10 mm showing region of interest by black box. **(B)** PKH26-labeled MHP36 and MHP36-Dax1KD co-localized with synaptophysin positive staining at 28 days post MCAO (scale bar = 5 μm; region of interest is red box in schema of coronal level in **A**), with co-localization shown in enlarged images. **(C)** Graphical representation of mean average intensity of synaptophysin in all experimental groups (**p < 0.01; ***p < 0.001 ipsilateral (I) vs. contralateral (C); ^###^*p* < 0.001 vs. vehicle in ipsilateral hemisphere; ^δ^*p* < 0.05 MHP36 vs. MHP36-Dax1KD); two-way ANOVA, *post-hoc* Bonferroni’s test to correct for multiple comparisons (*n* = 3 all groups).

**Figure 6:**
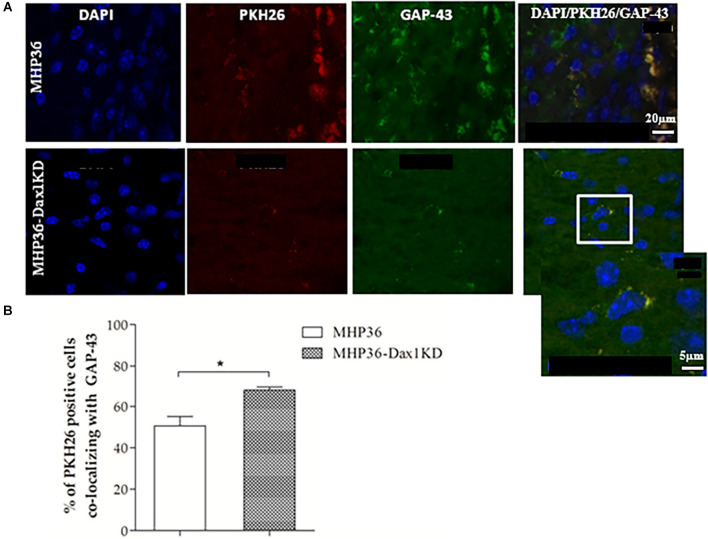
MHP36-Dax1KD grafts increased expression of GAP-43 in the host brain. **(A)** MHP36-Dax1KD and MHP36 grafts expressed the marker for synaptic plasticity GAP-43 (scale bar = 20 μm, Bregma: -0.3 ± 0.10 mm) and at higher magnification, PKH26 labeled MHP36-Dax1KD co-localizing with GAP-43 (white inset; scale bar = 5 μm). **(B)** Percentage of PKH26-labeled cells co-localizing with GAP-43 in the cortex at 28 days post-MCAO in animals grafted with MHP36 and MHP36-Dax1KD (*p < 0.05; *n* = 3 all groups).

**Figure 7:**
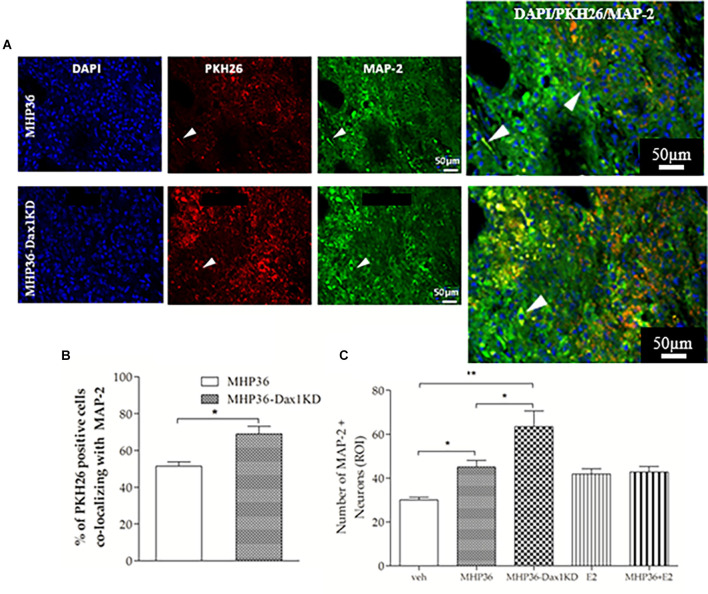
MHP36-Dax1KD grafts promoted neuronal differentiation in the host brain. **(A)** Peri-infarct cortex in mice receiving the PKH26 labeled MHP36 and MHP36-Dax1KD stained with MAP-2 (scale bar = 50 μm, Bregma: -0.3 ± 0.10 mm), with co-localization shown in enlarged images. **(B)** Percentage of PKH26 positive cells co-localizing with MAP-2 in the MHP36 and MHP36-Dax1KD grafted animals. **(C)** Number of MAP-2 stained neurons across all groups (*p < 0.05, **p < 0.01; *n* = 3 all groups).

**Figure 8:**
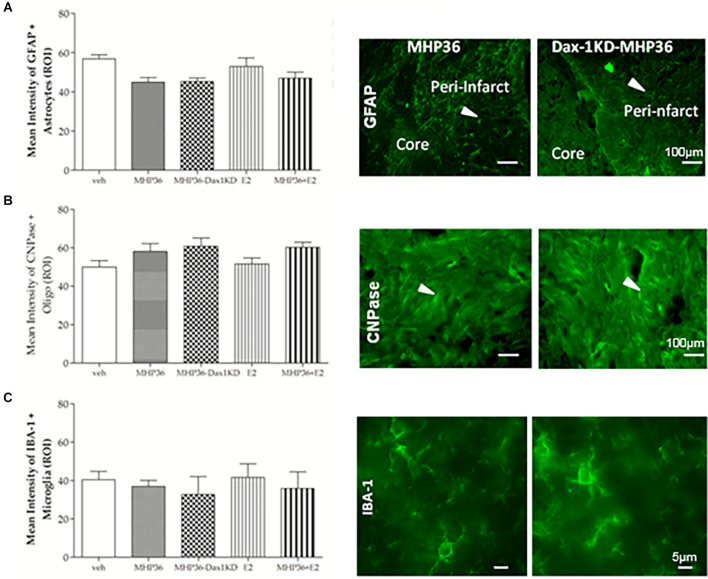
Mean intensity staining for markers of astrocytes, oligodendrocytes, and microglia. Graphical representation of mean average intensity staining in the peri-infarct cortex for **(A)** GFAP (astrocytes), **(B)** CNPase (oligodendrocytes), and **(C)** IBA-1 (microglia) markers in all experimental groups, with representative images from MHP36 and MHP36-Dax1KD on right. There was no statistical difference in expression profiles across the experimental groups; *n* = 3.

At 28 days post-MCAO, there was a significant decrease in lesion volume in MHP36-Dax1KD grafted mice (**12.0 ± 1.66 mm^3^**) compared to vehicle (18.5 ± 1.34 mm^3^, *p < 0.01; Figures 4A–C). At the time of MCAO (prior to transplantation) all animals showed a similar reduction in cerebral blood flow (≥85%) and similar reperfusion post-occlusion (Figure 4D). Hence, differences in infarcts were not attributed to differences in blood flow reduction or reperfusion as a result of the MCAO procedure. Using the mouse anti-synaptophysin antibody, a significant increase in mean intensity was observed in MHP36-Dax1KD grafted mice compared to MHP36 (Figure 5). PKH26/GAP-43 co-localized cells were observed in the peri-lesion cortex with a significant increase in GAP-43 expression in MHP36-Dax1KD mice compared to MHP36 (Figure 6). Significant increases in MAP-2/PKH26 co-localizing cells (Figures 7A,B) and the number of MAP-2^+ve^cells in peri-infarct cortex were observed in MHP36-Dax1KD compared to MHP36 transplanted mice (Figure 7C), whereas no differences in GFAP, CNPase, or IBA-1^+ve^ staining amongst groups were observed (Figure 8).

## Discussion

The key findings of the present study are that: (1) Dax-1 knockdown increased 17β-estradiol secretion from NSCs and; (2) MHP36-Dax1KD transplants enhanced the functional recovery and reduced infarct volume *in vivo* after experimental stroke.

We used Dax-1 knockdown to overexpress 17β-estradiol in NSCs, given that Dax-1 functions as a repressor of trans-activation of the aromatase gene. Indeed, we showed that MHP36-Dax1KD cells exhibited increased aromatase overexpression and increased 17β-estradiol production. This is fitting with a previous study where targeted disruption of *Dax-1* resulted in aromatase overexpression (Wang et al., 2001) with no alterations in the expression of other proteins (e.g., StAR and Cyp11a). Immunostaining and RT-PCR indicated that MHP36-Dax1KD cells may exhibit increased expression of ERβ and ERα but not GPR30 which is consistent with the finding that Dax-1 acts as a repressor of nuclear receptors (Jadhav et al., 2011). Finally, MHP36-Dax1KD cells secreted increased 17β-estradiol by 2.5 fold compared to MHP36 cells *in vitro*. As far as we are aware, this is the first study to identify Dax-1 knockdown as a useful target to increase 17β-estradiol secretion from stem cells. This strategy could be exploited therapeutically for diseases where 17β-estradiol is beneficial including stroke.

We, therefore, interrogated the application of 17β-estradiol overexpressing NSCs in a relevant disease model *in vivo*. Indeed, we found that MHP36-Dax1KD cells enhanced functional recovery and reduced lesion size *in vivo* after experimental stroke. Whilst Dax-1 regulates a variety of other functions relevant to stroke (e.g., progesterone and androgen receptors; Jadhav et al., 2011), we attribute, at least in part, these *in vivo* effects to the local production of 17β-estradiol of the implanted cells as these manipulated cells have increased 17β-estradiol production when cultured *in vitro*. There is a wealth of studies showing 17β-estradiol improves stroke outcomes (for reviews see Hurn and Macrae, 2000; Etgen et al., 2011) including improved functional recovery after stroke (Li et al., 2010).

We found that functional recovery was accelerated and enhanced in MHP36-Dax1KD mice. This is important because functional recovery after stroke in humans is slow and often incomplete. In the foot fault test, MHP36-Dax1KD transplants not only completely restored limb function to pre-MCAO baseline scores by 28 days post-MCAO but also accelerated recovery of limb function to 14 days post-MCAO. In the cylinder test, MHP36-Dax1KD completely restored symmetry to pre-MCAO scores by 28 days post-MCAO supporting our foot fault test results. However, the preferred use of the contralateral (“impaired”) paw in the unmodified MHP36 and MHP36+E_2_ groups is a surprising result. In order to understand this result, we compared synaptic plasticity in ipsilateral vs. contralateral hemispheres. Neuronal connections are continuously remodeled and suffer intense adaptive functional and structural reorganization after lesions (Carmichael, 2003; Rossi et al., 2007; Giraldi-Guimaraes et al., 2009). In line with other studies showing 17β-estradiol increases synaptogenesis (Sellers et al., 2015), we observed increased synaptogenesis in the ipsilateral compared to the contralateral hemisphere, which could explain the preferred use of the contralateral vs. ipsilateral paw. However, increased ipsilateral synaptogenesis was observed across the treatment groups and was especially enhanced in the MHP36-Dax1KD group (vs. the unmodified MHP36 group) where there was no opposite asymmetry. Enhanced synaptogenesis in MHP36-Dax1KD vs. MHP36 grafted mice was supported by a significant increase in the percentage of MHP36-Dax1KD cells co-localized with GAP-43, a presynaptic marker of axonal and synaptic growth. In addition, co-localization with neuronal marker MAP-2 indicated increased neuronal differentiation in the MHP36-Dax1KD vs. unmodified NSC grafted animals, whereas lack of changes in GFAP and CNPase staining amongst groups possibly indicate grafted cells did not differentiate into astrocytes or oligodendrocytes, respectively. In summary, based on two behavioral tests, chosen for their ability to detect deficits at chronic time-points, overexpressing 17β-estradiol NSCs improved functional recovery to pre-MCAO baseline levels, possibly mediated, at least in part, by inducing more intensive plastic changes.

We were surprised that lesion size was significantly reduced by MHP36-Dax1KD 48 h post-MCAO. This is of particular interest, as the therapeutic window of 17β-estradiol neuroprotection has been reported to be narrow (6 h) in previous studies (Liu et al., 2007; Lu et al., 2020). Given the challenges of early time to hospital presentation for acute stroke patients render many ineligible for thrombolytic therapy, extending the therapeutic window of neuroprotection to 48 h in the present study is a novel and important finding and is possibly mediated by beneficial effects on secondary damage due to the presence of sustained exposure to high levels of local cellular production of 17β-estradiol at the lesion site. This would explain the lack of neuroprotection by 17β-estradiol alone (no stem cells) as those animals would be exposed to only transient levels of 17β-estradiol, given that 17β-estradiol is highly diffusible and metabolizes quickly in the injured brain (Lidin et al., 2022). Whist 17β-estradiol has been previously been shown to promote the efficacy of cell therapy by pre-conditioning or co-administration systemically (Liang et al., 2016; Yuan et al., 2018), further studies would be required to confirm the role of local cellular production of 17β-estradiol in the present study, possibly by blocking 17β-estradiol synthesis or antagonizing the response. Also worthy of consideration is whether the cellular production of 17β-estradiol stimulates additional growth factor synthesis from NSCs which could play a role in the beneficial effects. Nevertheless, as far as we are aware, this is the first study that has shown 17β-estradiol neuroprotection as long as 48 h after stroke. Therefore, administering cells that synthesize and deliver17β-estradiol into the lesion site at a relatively late time (48 h) after the ischemic event could be a clinically relevant strategy.

Taking advantage of stem cells’ migratory properties to deliver neuroprotective/regenerative agents locally to the site of brain injury is not a new concept (Chen et al., 2016) and is appealing to circumvent systemic adverse effects. Additionally, a lack of difference in the astrocyte marker GFAP and microglia/macrophage marker IBA-1amongst groups could indicate that there is no change in reactive gliosis or inflammation in response to the grafted cells. In addition, genetic modification of NSCs to enhance their mechanism of action is a viable strategy for stroke (Korshunova et al., 2020). Furthermore, targeting aromatase to increase local 17β-estradiol production in stroke is a conceivable approach, given that aromatase expression is increased in the ischemic penumbra in female hypertensive rats (Carswell et al., 2005) and in the serum of acute female stroke patients (Manwani et al., 2021). Alternatively, to preclude estrogenic systemic adverse effects, non-feminizing estrogen compounds have been designed and, like the present study, would be applicable to both sexes (Engler-Chiurazzi et al., 2017).

## Conclusion

In summary, we have formulated an NSC line with a superior therapeutic efficacy by manipulating Dax-1. We have shown for the first time that Dax-1 knockdown is a good target to increase 17β-estradiol secretion from stem cells and have proven their utility in an *in vivo* disease model for stroke. Of particular interest is that the therapeutic window of 17β-estradiol neuroprotection can be extended from 6 h shown in previous studies to 48 h after stroke. In addition, functional recovery after stroke was not only enhanced but also accelerated. Therefore, additional and sustained, rather than brief, exposure to 17β-estradiol in the face of the ischemic insult is a strategy that should be further explored to allow us to extend the therapeutic window, aid repair, and maximize recovery after stroke.

## Data Availability Statement

The datasets presented in this study can be found in online repositories. The names of the repository/repositories and accession number(s) can be found below: Data underpinning this publication are openly available from the University of Strathclyde KnowledgeBase at: https://doi.org/10.15129/fafa972b-0ef9-434c-911a-895796574a12.

## Ethics Statement

The animal study was reviewed and approved by UK Animals (Scientific) Procedures Act (1986) with approval by the Home Office of the United Kingdom (Project License number 60/4469) and Ethical Review of the University of Strathclyde.

## Author Contributions

SP performed the experiments, analyzed and interpreted the datasets and wrote the draft manuscript. DU generated the MHP36-Dax1KD cells using the lentiviral vector system for silencing *Dax-1* in MHP36 cells. MM provided MHP36 cells and advised on experimental design and interpretation of the results. RT advised on experimental design for several experiments including PCR. RP advised on experimental design, co-supervised the project and contributed to the interpretation of the results. HC conceived the study, supervised the project and wrote the manuscript with support from other authors. All authors contributed to the article and approved the submitted version.

## Conflict of Interest

The authors declare that the research was conducted in the absence of any commercial or financial relationships that could be construed as a potential conflict of interest.

## Publisher’s Note

All claims expressed in this article are solely those of the authors and do not necessarily represent those of their affiliated organizations, or those of the publisher, the editors and the reviewers. Any product that may be evaluated in this article, or claim that may be made by its manufacturer, is not guaranteed or endorsed by the publisher.

## Abbreviations

E2: 17β-estradiol
MHP36: Maudsley hippocampal murine neural progenitor cell line clone 36
Dax-1: Dosage-sensitive sex reversal, adrenal hypoplasia critical region, on chromosome X, gene 1
MHP36-Dax1KD: Dax-1 knock-down MHP36 cell line
MCAO: transient middle cerebral artery occlusion.

## References

[B1] Bingham D. Macrae I. M. Carswell H. V. (2005). Detrimental effects of 17β-oestradiol after permanent middle cerebral artery occlusion. J. Cereb. Blood Flow Metab. 25, 414–420. 10.1038/sj.jcbfm.9600031 15647739

[B2] Carmichael S. T. (2003). Plasticity of cortical projections after stroke. Neuroscientist 9, 64–75. 10.1177/1073858402239592 12580341

[B5] Carswell H. V. Bingham D. Wallace K. Nilsen M. Graham D. I. Dominiczak A. F.. (2004). Differential effects of 17β-oestradiol on stroke damage in stroke prone (SHRSP) and normotensive rats. J. Cereb. Blood Flow Metab. 24, 298–304. 10.1097/01.wcb.0000112322.75217.fd 15091110

[B4] Carswell H. V. O. Dominiczak A. F. Garcia-Segura L. M. Harada N. Hutchison J. B. Macrae I. M. (2005). Brain aromatase expression after experimental stroke: topography and time course. J. Steroid Biochem. Mol. Biol. 96, 89–91. 10.1016/j.jsbmb.2005.02.016 15896953

[B3] Carswell H. V. Macrae I. M. Farr T. D. (2009). Complexities of oestrogen in stroke. Clin. Sci. (Lond) 118, 375–389. 10.1042/CS20090018 20001955

[B6] Chen B. Zhang F. Li Q. Y. Gong A. Lan Q. (2016). Protective effect of Ad-VEGF-bone mesenchymal stem cells on cerebral infarction. Turk. Neurosurg. 26, 8–15. 10.5137/1019-5149.JTN.11488-14.3 26768863

[B7] Damian C. Ghuman H. Mauney C. Azar R. Reinartz J. Badylak S. F.. (2021). Post-stroke timing of ECM hydrogel implantation affects biodegradation and tissue restoration. Int. J. Mol. Sci. 22:11372. 10.3390/ijms222111372 34768800 PMC8583606

[B8] Engler-Chiurazzi E. B. Covey D. F. Simpkins J. W. (2017). A novel mechanism of non-feminizing estrogens in neuroprotection. Exp. Gerontol. 94, 99–102. 10.1016/j.exger.2016.10.013 27818250 PMC5415429

[B9] Etgen A. M. Jover-Mengual T. Zukin R. S. (2011). Neuroprotective actions of estradiol and novel estrogen analogs in ischemia: translational implications. Front. Neuroendocrinol. 32, 336–352. 10.1016/j.yfrne.2010.12.005 21163293 PMC3080451

[B10] Giraldi-Guimaraes A. Rezende-Lima M. Bruno F. P. Mendez-Otero R. (2009). Treatment with bone marrow mononuclear cells induces functional recovery and decreases neurodegeneration after sensorimotor cortical ischemia in rats. Brain Res. 1266, 108–120. 10.1016/j.brainres.2009.01.062 19368806

[B11] Gray J. A. Grigoryan G. Virley D. Patel S. Sinden J. D. Hodges H. (2000). Conditionally immortalized, multipotential and multifunctional neural stem cell lines as an approach to clinical transplantation. Cell Transplant. 9, 153–168. 10.1177/096368970000900203 10811390

[B12] Guo H. Liu M. Zhang L. Wang L. Hou W. Ma Y.. (2020). The critical period for neuroprotection by estrogen replacement therapy and the potential underlying mechanisms. Curr. Neuropharmacol. 18, 485–500. 10.2174/1570159X18666200123165652 31976839 PMC7457406

[B13] Hermann D. M. Peruzzotti-Jametti L. Schlechter J. Bernstock J. D. Doeppner T. R. Pluchino S. (2014). Neural precursor cells in the ischemic brain - integration, cellular crosstalk and consequences for stroke recovery. Front. Cell. Neurosci. 8:291. 10.3389/fncel.2014.00291 25278840 PMC4165213

[B14] Hodis H. N. Mack W. J. Henderson V. W. Shoupe D. Budoff M. J. Hwang-Levine J.. (2016). Vascular effects of early versus late postmenopausal treatment with estradiol. N. Engl. J. Med. 374, 1221–1231. 10.1056/NEJMoa1505241 27028912 PMC4921205

[B15] Horsburgh K. Macrae I. M. Carswell H. (2002). Estrogen is neuroprotective via an apolipoprotein E-dependent mechanism in a mouse model of global ischemia. J. Cereb. Blood Flow Metab. 22, 1189–1195. 10.1097/01.wcb.0000037991.07114.4e 12368657

[B16] Hurn P. D. Macrae I. M. (2000). Estrogen as a neuroprotectant in stroke. J. Cereb. Blood Flow Metab. 20, 631–652. 10.1097/00004647-200004000-00001 10779008

[B17] Jadhav U. Harris R. M. Jameson J. L. (2011). Hypogonadotropic hypogonadism in subjects with dax1 mutations. Mol. Cell. Endocrinol. 346, 65–73. 10.1016/j.mce.2011.04.017 21672607 PMC3185185

[B18] Korshunova I. Rhein S. García-González D. Stölting I. Pfisterer U. Barta A.. (2020). Genetic modification increases the survival and the neuroregenerative properties of transplanted neural stem cells. JCI Insight 5:e126268. 10.1172/jci.insight.126268 31999645 PMC7101138

[B19] Lee J. M. Bae J. S. Jin H. K. (2010). Intracerebellar transplantation of neural stem cells into mice with neurodegeneration improves neuronal networks with functional synaptic transmission. J. Vet. Med. Sci. 72, 999–1009. 10.1292/jvms.09-0514 20339259

[B21] Li W. Lu P. Lu Y. Wei H. Niu X. Xu J.. (2020). 17β-estradiol protects neural stem/progenitor cells against ketamine-induced injury through estrogen receptor β pathway. Front. Neurosci. 14:576813. 10.1016/0006-2952(75)90018-0 33100963 PMC7556164

[B20] Li J. Siegel M. Yuan M. Zeng Z. Finnucan L. Persky R.. (2010). Estrogen enhances neurogenesis and behavioral recovery after stroke. J. Cereb. Blood Flow Metab. 31, 413–425. 10.1038/jcbfm.2010.181 20940729 PMC3049534

[B22] Liang C. C. Liu H. L. Chang S. D. Chen S. H. Lee T. H. (2016). The protective effect of human umbilical cord blood CD34+ cells and estradiol against focal cerebral ischemia in female ovariectomized rat: cerebral MR imaging and immunohistochemical study. PLoS One 11:e0147133. 10.1371/journal.pone.0147133 26760774 PMC4711929

[B23] Lidin E. Sköld M. K. Angéria M. Davidsson J. Risling M. (2022). Hippocampal expression of cytochrome P450 1B1 in penetrating traumatic brain injury. Int. J. Mol. Sci. 23:722. 10.3390/ijms23020722 35054909 PMC8775891

[B24] Liu R. Wang X. Liu Q. Yang S. H. Simpkins J. W. (2007). Dose dependence and therapeutic window for the neuroprotective effects of 17beta-estradiol when administered after cerebral ischemia. Neurosci. Lett. 415, 237–241. 10.1016/j.neulet.2007.01.074 17331646 PMC1936945

[B25] Lu Y. Sareddy G. R. Wang J. Zhang Q. Tang F. L. Pratap U. P.. (2020). Neuron-derived estrogen is critical for astrocyte activation and neuroprotection of the ischemic brain. J. Neurosci. 40, 7355–7374. 10.1523/JNEUROSCI.0115-20.2020 32817249 PMC7534920

[B26] Manwani B. Fall P. Zhu L. O’Reilly M. R. Conway S. Staff I.. (2021). Increased P450 aromatase levels in post-menopausal women after acute ischemic stroke. Biol. Sex Differ. 12:8. 10.1186/s13293-020-00357-w 33413673 PMC7792154

[B27] Metz G. A. Whishaw I. Q. (2002). Cortical and subcortical lesions impair skilled walking in the ladder rung walking test: a new task to evaluate fore- and hindlimb stepping, placing and co-ordination. J. Neurosci. Methods 115, 169–179. 10.1016/s0165-0270(02)00012-2 11992668

[B28] Mihai M. C. Popa M. A. Suică V. I. Antohe F. Jackson E. K. Leeners B.. (2021). Proteomic analysis of estrogen-mediated enhancement of mesenchymal stem cell-induced angiogenesis in vivo. Cells 10:2181. 10.3390/cells10092181 34571830 PMC8468955

[B29] Modo M. Stroemer R. P. Tang E. Patel S. Hodges H. (2002). Effects of implantation site of stem cell grafts on behavioral recovery from stroke damage. Stroke 33, 2270–2278. 10.1161/01.str.0000027693.50675.c5 12215598

[B30] Muir K. W. Bulters D. Willmot M. Sprigg N. Dixit A. Ward N.. (2020). Intracerebral implantation of human neural stem cells and motor recovery after stroke: multicentre prospective single-arm study (PISCES-2). J. Neurol. Neurosurg. Psychiatry 91, 396–401. 10.1136/jnnp-2019-322515 32041820 PMC7147186

[B31] Nicholls F. J. Liu J. R. Modo M. (2017). A comparison of exogenous labels for the histological identification of transplanted neural stem cells. Cell Transplant. 26, 625–645. 10.3727/096368916X693680 27938486 PMC5661216

[B32] Patkar S. Tate R. Modo M. Plevin R. Carswell H. V. (2012). Conditionally immortalised neural stem cells promote functional recovery and brain plasticity after transient focal cerebral ischaemia in mice. Stem Cell Res. 8, 14–25. 10.1016/j.scr.2011.07.001 22099017

[B33] Patkar S. Tate R. Modo M. Plevin R. Carswell H. V. O. (2009). Characterisation of neural stem cells for oestrogen *in vitro*: potential for improving stem cell based therapy for stroke. J. Cereb. Blood Flow Metab. 29, S544–S552. 10.1038/jcbfm.2009.168

[B34] Patkar S. (2010). Effects of oestrogen on neural stem cell success in a stroke model. Doctoral Thesis. Available online at: https://pureportal.strath.ac.uk/en/publications/effects-of-oestrogen-on-neural-stem-cell-success-in-a-stroke-mode.

[B35] Percie du Sert N. Hurst V. Ahluwalia A. Alam S. Avey M. T. Baker M.. (2020). The ARRIVE guidelines 2.0: updated guidelines for reporting animal research. PLoS Biol. 18:e3000410. 10.1371/journal.pbio.3000410 32663219 PMC7360023

[B36] Ransohoff R. M. (2007). The MHP36 line of murine neural stem cells expresses functional CXCR1 chemokine receptors that initiate chemotaxis in vitro. J. Neuroimmunol. 186:199. 10.1016/j.jneuroim.2007.03.018 17482680

[B37] Rewell S. S. Churilov L. Sidon T. K. Aleksoska E. Cox S. F. Macleod M. R.. (2017). Evolution of ischemic damage and behavioural deficit over 6 months after MCAo in the rat: selecting the optimal outcomes and statistical power for multi-centre preclinical trials. PLoS One 12:e0171688. 10.1371/journal.pone.0171688 28182727 PMC5300105

[B38] Rossi F. Gianola S. Corvetti L. (2007). Regulation of intrinsic neuronal properties for axon growth and regeneration. Prog. Neurobiol. 81, 1–28. 10.1016/j.pneurobio.2006.12.001 17234322

[B39] Schallert T. Fleming S. M. Leasure J. L. Tillerson J. L. Bland S. T. (2000). CNS plasticity and assessment of forelimb sensorimotor outcome in unilateral rat models of stroke, cortical ablation, parkinsonism and spinal cord injury. Neuropharmacology 39, 777–787. 10.1016/s0028-3908(00)00005-8 10699444

[B40] Schneider C. A. Rasband W. S. Eliceiri K. W. (2012). NIH image to imageJ: 25 years of image analysis. Nat. Methods 9, 671–675. 10.1038/nmeth.2089 22930834 PMC5554542

[B41] Sellers K. J. Erli F. Raval P. Watson I. A. Chen D. Srivastava D. P. (2015). Rapid modulation of synaptogenesis and spinogenesis by 17β-estradiol in primary cortical neurons. Front. Cell. Neurosci. 9:137. 10.3389/fncel.2015.00137 25926772 PMC4396386

[B42] Sinden J. D. Rashid-Doubell F. Kershaw T. R. Nelson A. Chadwick A. Jat P. S.. (1997). Recovery of spatial learning by grafts of a conditionally immortalized hippocampal neuroepithelial cell line into the ischaemia-lesioned hippocampus. Neuroscience 81, 599–608. 10.1016/s0306-4522(97)00330-8 9316014

[B43] Sohrabji F. (2015). Estrogen-IGF-1 interactions in neuroprotection: ischemic stroke as a case study. Front. Neuroendocrinol. 36, 1–14. 10.1016/j.yfrne.2014.05.003 24882635 PMC4247812

[B44] Strom J. O. Theodorsson A. Theodorsson E. (2009). Dose-related neuroprotective versus neurodamaging effects of estrogens in rat cerebral ischemia: a systematic analysis. J. Cereb. Blood Flow Metab. 29, 1359–1372. 10.1038/jcbfm.2009.66 19458604

[B45] Strom J. O. Theodorsson A. Theodorsson E. (2011). Mechanisms of estrogens’ dose-dependent neuroprotective and neurodamaging effects in experimental models of cerebral ischemia. Int. J. Mol. Sci. 12, 1533–1562. 10.3390/ijms12031533 21673906 PMC3111617

[B46] Wang Z. J. Jeffs B. Ito M. Achermann J. C. Yu R. N. Hales D. B.. (2001). Aromatase (Cyp19) expression is up-regulated by targeted disruption of Dax1. Proc. Natl. Acad. Sci. U S A 98, 7988–7993. 10.1073/pnas.141543298 11427738 PMC35455

[B47] Wassertheil-Smoller S. Hendrix S. L. Limacher M. Heiss G. Kooperberg C. Baird A.. (2003). Effect of estrogen plus progestin on stroke in postmenopausal women: the Women’s health initiative: a randomized trial. JAMA 289, 2673–2684. 10.1001/jama.289.20.2673 12771114

[B48] Yuan Z. Kang L. Wang Z. Chen A. Zhao Q. Li H. (2018). 17β-estradiol promotes recovery after myocardial infarction by enhancing homing and angiogenic capacity of bone marrow-derived endothelial progenitor cells through ERα-SDF-1/CXCR4 crosstalking. Acta Biochim. Biophys. Sin. (Shanghai) 50, 1247–1256. 10.1093/abbs/gmy127 30371725

[B49] Zhang X. L. Zhang X. G. Huang Y. R. Zheng Y. Y. Ying P. J. Zhang X. J.. (2021). Stem cell-based therapy for experimental ischemic stroke: a preclinical systematic review. Front. Cell. Neurosci. 15:628908. 10.3389/fncel.2021.628908 33935650 PMC8079818

